# Complete biochemical response after pulmonary metastasectomy in prostate adenocarcinoma

**DOI:** 10.1186/s40164-017-0085-2

**Published:** 2017-09-15

**Authors:** Jonathan Rush, Reet Pai, Rahul A. Parikh

**Affiliations:** 1 University of Pittsburgh Medical Center, Pittsburgh, PA USA; 2 University of Pittsburgh Department of Pathology, Pittsburgh, PA USA; 3 University of Pittsburgh Cancer Institute, Pittsburgh, PA USA

**Keywords:** Prostate adenocarcinoma, Pulmonary metastasis, Metastasectomy

## Abstract

**Background:**

Prostate cancer most commonly metastasizes to bones or lymph nodes, and metastatic prostate cancer is suggestive of disseminated disease. Metastatic disease is usually not amenable to surgery.

**Case presentation:**

The current report presents a unique case of in which the excision of a solitary pulmonary metastasis resulted in undetectable prostate-specific antigen.

**Conclusion:**

This case and others suggest metastasectomy could be considered in cases with solitary metastasis, and physicians should have a careful discussion regarding risks and possible benefits from surgical excision.

## Background

Prostate adenocarcinoma most commonly metastasizes to bones and regional lymph nodes. Visceral involvement in the form of pulmonary or hepatic disease is uncommon, and usually occurs after development of castrate resistant prostate cancer (CRPC).

Isolated pulmonary metastasis from prostate adenocarcinoma is extremely rare. In a large case series of 1290 patients with prostate adenocarcinoma, only 3.6% (47 patients) were identified with radiographic pulmonary metastases. Only 11 patients (0.9%) were noted to have solitary pulmonary metastases in the absence of disease at other sites [1]. In comparison, the incidence of pulmonary metastases seen at autopsy is much higher and ranges from 20 to 74%. In one such study, of 1367 patients with metastatic prostate cancer, 49.1% had pulmonary metastasis. In this series, 4 patients (0.03%) had isolated metastases to the lung while bone and lymph node involvement were much more common accounting for nearly 68 and 66% of all patients respectively [2].

## Case presentation

**Fig. 1:**
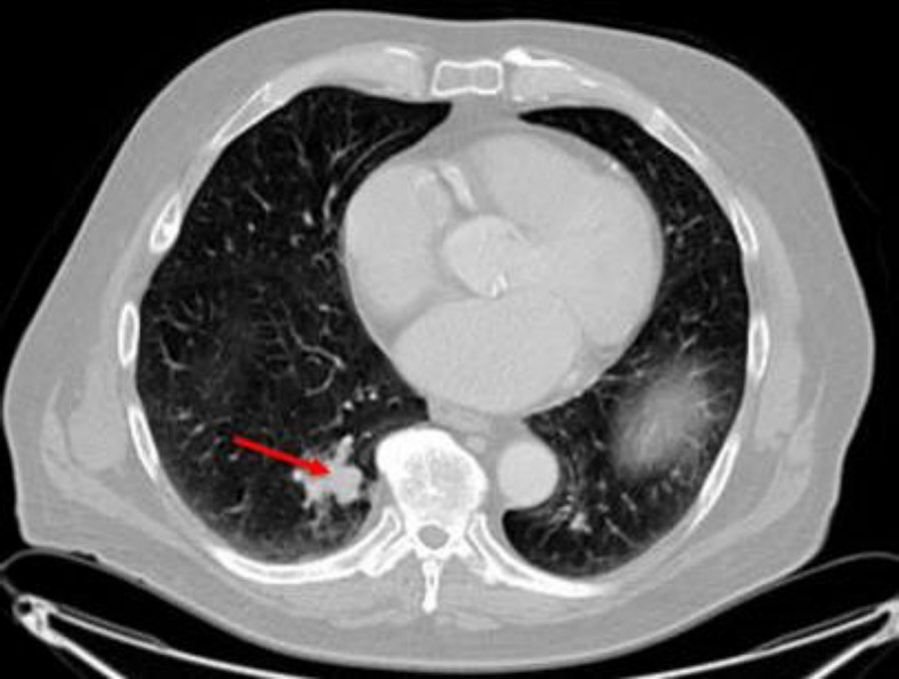
CT scan of chest. Red arrow indicates lung metastasis. PET scan and CT chest, abdomen and pelvis were only positive for the isolated lung metastasis

**Fig. 2:**
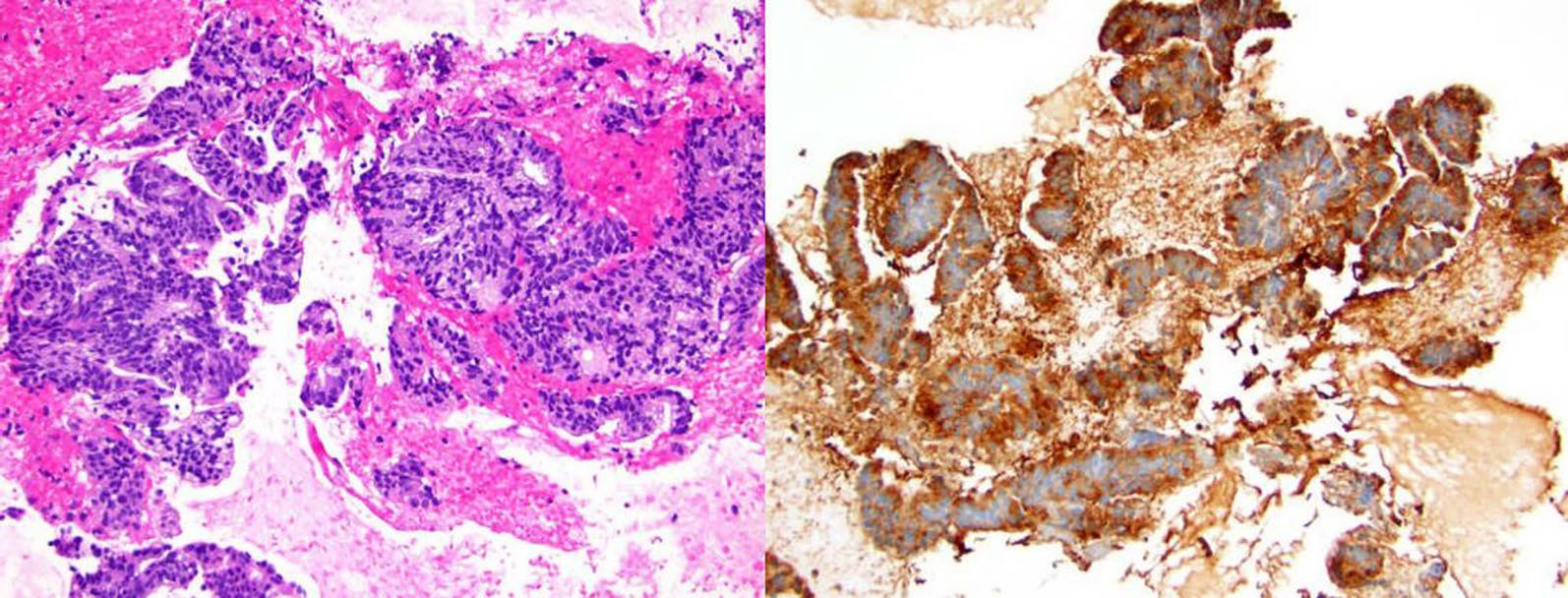
**a** (left) Hematoxylin and eosin stained cell block from nodule. This specimen reveals groups of malignant tumor cells with vacuolated cytoplasm consistent with prostate adenocarcinoma. **b** (right) Immunohistochemical stains were strongly positive for prostate-specific antigen (above) as well as NKX3.1, androgen receptor, and prostatic-specific acid phosphatase confirming prostatic origin

A 70-year-old man with no co-morbidities and an excellent Eastern Cooperative Oncology Group (ECOG) performance status of 0, presented with an elevated prostate-specific antigen (PSA) of 20 ng/mL on yearly examination. He underwent definitive surgery with radical prostatectomy without complications. Final pathology was consistent with Gleason 4 + 4, prostate adenocarcinoma, acinar-type, invading the submucosa, with perineural invasion but absent lymphovascular invasion. The tumor involved the neck of the bladder and resection margins were negative. His pathological stage at diagnosis was T4N0M0, thus localized stage IV disease. Thirteen dissected lymph nodes were absent for metastases. He completed adjuvant radiotherapy to his prostate bed and regional lymph node basin for T4 disease. His PSA nadired at 0.2 ng/mL post-operatively but rose to be 0.5 ng/mL, 16 months after radical prostatectomy. Subsequently, his PSA increased to 2.9 ng/mL over a period of 23 months with a PSA doubling time of 10 months. CT chest, abdomen and pelvis were notable for a pulmonary lesion measuring 4.7 cm × 3.2 cm (Fig. 1). A Fludeoxyglucose (^18^F) (FDG) PET-CT scan revealed the prior observed single pulmonary mass without evidence of skeletal involvement. Biopsy of this pulmonary lesion was performed and pathologic and immune-histochemical examination confirmed the diagnosis of prostate adenocarcinoma (Fig. 2a, b). After discussion of the risks of surgery versus systemic androgen deprivation therapy, a decision was made to proceed with surgical metatasectomy. A thoracoscopic segmentectomy was performed and revealed metastatic prostate adenocarcinoma with negative surgical margins. The patient’s PSA became undetectable following the surgery and has remained undetectable at 12- and 24-month follow up.

## Discussion

Patients with a high Gleason score prostate adenocarcinoma have a high risk of both localized and systemic metastatic disease after definitive therapy. The standard of care for these patients with high-risk disease after developing biochemical recurrence or development of new metastatic disease is surgical or chemical castration. Systemic therapy is preferred due to the high risk of micro-metastatic disease. However, androgen deprivation therapy (ADT) is associated with a number of side-effects including sexual dysfunction [3], osteoporosis and bone fracture, anemia [4], vasomotor symptoms, fatigue [5], and potential cardiovascular harm [6]. In patients with metastatic prostate adenocarcinoma, systemic therapy with ADT is the standard of care. ADT is palliative and not curative, and generally these patients continue ADT at progression, which can result in cumulative toxicity from the use of ADT over a number of years.

Long-term survival after surgical resection of pulmonary metastasis has been described for multiple malignancies including colorectal cancer [7], soft tissue sarcomas [8], malignant melanoma [9], and head and neck cancer [10]. For colo-rectal malignancies, resection of pulmonary metastases was associated with improvement in progression free and overall survival [11]. It is possible that survival may be extended for cases of isolated metastatic prostate cancer by metastasectomy. We reviewed the literature and found reports of prostate adenocarcinoma metastasectomy leading to a complete response. One case describes a 69 year-old male with a rising PSA after initial radical prostatectomy. He had a single 1.2 cm nodule treated with pulmonary wedge resection. His PSA level dropped and remained undetectable after 12 years of follow up without requiring any additional therapy [12]. Another report revealed a 72 year-old male treated with radical prostatectomy and bilateral pelvic lymph node dissection with Gleason 3 + 3. He had a rising PSA, and CT revealed a pulmonary nodule. He had a wedge resection, and his PSA fell to undetectable levels at 3-year follow up without any other therapy [13].

The indication and role for metastasectomy in patients with high-risk prostate adenocarcinoma is unclear. In other cancers, the number of lung metastasis [14], presence of lymph node metastasis [15], and elevated serum markers such as serum carcinoembryonic antigen are known to influence the outcome [16]. Many of these features are important to the clinical course of prostate cancer, and similarly, these features could be considered in the decision for surgical intervention. Prior to metastasectomy, it is reasonable to rule out additional sites of metastatic disease using a FDG PET-CT scan or a technetium-99 m bone scan along with a conventional CT scan of the chest, abdomen and pelvis. Our patient with high risk Gleason 4 + 4 disease with a PSA of 20 ng/dL at diagnosis had an excellent response to metastasectomy. This is evidence that a solitary pulmonary metastasis may not equate to widely metastatic disease in prostate adenocarcinoma. Isolated pulmonary metastasis is rare, and pulmonary metastasectomy should be considered in a select group of patients with minimal co-morbidities and good overall performance status after discussion of risks and benefits of this approach. Criteria for pulmonary metastasectomy include adequate control of the primary tumor, the ability to resect all metastatic disease, the absence of additional sites of metastasis, and adequate cardiopulmonary reserve for the planned surgery [17]. Although pulmonary resection may or may not affect the natural history of the disease, the presented case suggests the possibility of achieving cure after pulmonary metastasectomy. The benefit of this approach is twofold, it reduces the burden of side-effects from use of ADT and it can potentially cure patients. Physicians who encounter a similar situation should have a careful discussion regarding the risks and possible benefits of surgical intervention.

## Conclusion

Our observations support the role of metastasectomy for isolated pulmonary metastases in prostate adenocarcinoma. Metastasectomy should be considered in selected patients with overall excellent performance status and life expectancy since it can delay or prevent the use of ADT and its side-effects in these patients.
